# Environmental Risk Assessment of Recycled Products of Spent Coppery Etchant in Jiangsu Province, China

**DOI:** 10.3390/ijerph18157881

**Published:** 2021-07-26

**Authors:** Xiaowei Xu, Jing Hua, Houhu Zhang, Zehua Zhao, Yi Wang, Dapeng Zhang, Jun Zhang, Xiaoxi Chen

**Affiliations:** Nanjing Institute of Environmental Science, Ministry of Ecology and Environment of China, Nanjing 210042, China; xuxiaowei@nies.org (X.X.); huajing@nies.org (J.H.); zhh@nies.org (H.Z.); wangyi@nies.org (Y.W.); zhangdapeng@nies.org (D.Z.); zhangjun@nies.org (J.Z.); chenxiaoxi@nies.org (X.C.)

**Keywords:** recycled product, spent coppery etchant, environmental risk assessment, heavy metals

## Abstract

With the vigorous development of the 5G industry, the characteristic hazardous waste, spent coppery etchant, was also produced in large quantities. In recent years, there are many companies that have begun to collect spent coppery etchant for the purpose of producing recycled products, such as copper sulfate, copper oxide, basic copper chloride, and copper powder, which often contain large amounts of heavy metals. However, due to the lack of relevant standards and applicable regulatory measures, some of the recycled products flow to the feed processing industry and even to the food processing industry. This study investigated the pollution status of heavy metals in recycled products of spent coppery etchant and evaluated the impact of recycled products exposure on human health. The results showed that the content of Zn was the highest, which was 21 times higher than the corresponding standard limit. Human health risk assessment indicated that the hazard quotients of As account for 87.5% of the entire HI value, while the average carcinogenic risk values of As for copper sulfate, copper oxide, basic copper chloride, and copper powder are 1.09 × 10^−5^, 3.19 × 10^−5^, 1.29 × 10^−5^, 7.94 × 10^−6^, respectively. Meanwhile, suggestions on the supervision of recycled products and the concentration limits of heavy metals in recycled products were put forward.

## 1. Introduction

With the vigorous development of China’s 5G industry, the production capacity of mobile phones, computers, and other electronic products has shown explosive growth [1]. At the same time, the production of spent copper etchant, as a waste fluid from the etching process in the electronic component manufacturing industry, is increasing year by year [2]. It is reported that Jiangsu Province, as one of the most crucial electronic products manufacturing areas in China, produces more than 350,000 tons of spent coppery etchant every year [3]. Spent coppery etchant is a kind of hazardous waste (HW22) listed on the National Hazardous Waste List and usually contains about 10% copper and other heavy metals in varying amounts, such as zinc, nickel, cadmium, chromium, and arsenic [4].

In recent years, there are numerous companies that have begun to collect spent coppery etchant for the purpose of producing recycled products. Comprehensive utilization methods of spent coppery etchant mainly include a synthetic process, electrolysis process, and replacement process. The synthesis method takes the form of extracting recycled copper products by neutralization of acidic (alkaline) copper that contains etching waste liquor or by a reaction with acid or alkali. The replacement method refers to the process of adding iron powder and aluminum powder into the spent coppery etchant to replace copper ions to produce recycled copper products. The electrolysis method takes the form of producing recycled copper products by electrolysis equipment using spent coppery etchant as raw material. The commonly recycled products are copper sulfate, basic copper carbonate, copper hydroxide, and copper powder. However, due to the limitation of production technology and enterprise management levels, these recycled products usually contain heavy metals such as Zn, Ni, Cd, Cr, and As [5].

At present, these recycled products are sold mainly in accordance with industrial product quality standards, such as Industrial Copper Sulfate (HG/T 5215, 2017), Copper Oxide Powder (GB/T 26046, 2010), Industrial Cuprous Chloride (GB/T 27562, 2011), Crude Copper (YS/T 70, 2005), Industrial Basic Copper Carbonate (HG/T 4825, 2015), Industrial Basic Copper Chloride (HG/T 4826, 2015), and Regenerated Copper Hydroxide (HG/T 4699, 2014). However, these product quality standards fail to take into account recycled products made from hazardous waste. Thus, they are not fully applicable to recycled products from spent copper etchants.

According to the investigation, the recycled products are used primarily for industrial purposes, including copper smelting raw materials, cables, wood preservatives, fungicides, etc., but some of the recycled products flow to the feed processing industry and even to the food processing industry [5]. Previous studies have established that heavy metals can cause anemia, bronchitis, and even damage to the nervous system [6] through ingestion, inhalation, and dermal contact [7]. For example, cadmium can replace calcium in the bone when it goes into the body, causing severe softening of the bones and also causing dysfunction of human organs [8]. Additionally, acute mercury poisoning can induce hepatitis and hematuria. Inhalation of chromium particles may irritate the respiratory tract and cause laryngitis and bronchitis [9]. As a result, the U.S. EPA has listed some metals as priority pollutants because of their toxicity, bioaccumulation, and low degradability [10].

Once these recycled products enter the feed processing industry or even the food processing industry, the heavy metals in the recycled products may harm human health through ingestion, skin contact, and inhalation. In recent years, some researchers have investigated the human health risks caused by various environmental pollution. However, most of them have concentrated on large-scale and regional pollution research and discussed the risks for large regions [11]. They are often not related to the health risks of specific industries, especially the electronic components manufacturing industry [6]. The output of recycled products from spent coppery etchant is increasing year by year, but its ecological risk is rarely concerned. Therefore, this work used the risk assessment model recommended by the U.S. EPA to investigate and evaluate the heavy metal pollution level of recycled products and estimate the impact of heavy metal exposure on human health.

## 2. Materials and Methods

### 2.1. Study Area

As an important manufacturing area of electronic products in China, the output of spent coppery etchant in Jiangsu Province accounts for 47.5% of the whole country. So far, there are more than 10 spent coppery etchant comprehensive utilization companies with an approved scale of more than 10,000 tons. In this study, the main recycled products of these large-scale enterprises were collected for detection and analysis.

### 2.2. Sample Collection and Analysis

The common products recovered from spent coppery etchant include mainly copper sulfate, copper oxide, basic copper chloride, and copper powder. In this investigation, 5 samples of each of the above products were collected, and a total of 20 samples of recycled products were collected. Each sample was collected by the quartering method, and each sample was about 2 kg. Then the samples were preserved and transported according to the Technical Specifications on Sampling and Sample Preparation from Industry Solid Waste (HJ/T 20, 1998). Before analysis, samples were air-dried, pulverized (TRM4-1L, Gredeman, Shanghai, China), and sieved (100 mesh).

For the determination of heavy metal content in each sample, 100 mg of completely mixed recycled product was digested with 10 ml of aqua regia and hydrofluoric acid mixture (7:3 *v*/*v*) followed by microwave digestion (Mars 5, CEM, Raleigh, NC, USA) [12]. Then, the concentrations of zinc, lead, nickel, cadmium, and chromium in the digestion were determined by inductively coupled plasma mass spectrometry (iCAP RQ, Thermo Fisher, Waltham, MA, USA), while mercury and arsenic were determined by atomic fluorescence spectrometry (AFS-8520, Haiguang, Beijing, China) [12,13].

In order to ensure the accuracy and reliability of the experimental data, the reagents used in the experiment are all analytically pure. Parallel samples and blank samples were set for the determination of heavy metals, and the results were within the allowable error range. The detection limits of zinc, nickel, cadmium, lead, chromium, mercury and arsenic in digestion solution were 0.01 mg/L, 0.02 mg/L, 0.01 mg/L, 0.03 mg/L, 0.02 mg/L, 0.0002 mg/L, and 0.0001 mg/L, respectively.

### 2.3. Comprehensive Index Method

The comprehensive index method [14] was used to evaluate the heavy metal pollution of recycled products, and the calculation formula used was Equation (1).

(1) $$P_{i}=\frac{1}{n}\sum^{\;}C_{i}/S_{i}$$

where *P* is the comprehensive index of heavy metals pollution status for recycled products (unitless), C is the concentration of metals in the recycled products (mg/kg), and S is the product quality standard value of heavy metals (mg/kg). Table 1 and Table 2 show the product quality standard value and product quality classification respectively.

### 2.4. Human Health Risk Assessment

It is reported that some recycled products have entered the feed processing industry and even the food processing industry, which may endanger human health through inhalation, ingestion, and skin contact. The general exposure equation used in this study is based on recommendations provided by the U.S. EPA. The average daily intake (ADI) can be calculated according to Equations (2)–(4).

(2) $${\mathrm{ADI}}_{ing}=C\times \frac{IngR\times EF\times ED}{BW\times AT}\times 10^{-6}$$

(3) $${\mathrm{ADI}}_{der}=C\times \frac{SA\times AF\times ABS\times EF\times ED}{BW\times AT}\times 10^{-6}$$

(4) $${\mathrm{ADI}}_{inh}=C\times \frac{InhR\times EF\times ED}{PEF\times BW\times AT}$$

where ADI_*ing*, ADI_*der*, and ADI_*inh* are the average daily intake from ingestion, inhalation, and dermal absorption respectively (mg/kg-day), *IngR* and *InhR* are the ingestion and inhalation rate of recycled products respectively (mg/day, m^3^/day), *BW* is the bodyweight of the exposed individual (kg), *PEF* is the emission factor (m^3^/kg), *AF* is the adherence factor (mg/cm^2^-day), *ABS* is the dermal absorption factor (unitless), *SA* is the exposed skin surface area (cm^2^), *C* is the concentration of metals in the recycled products (mg/kg), *ED* is the exposure duration (year), *EF* is the exposure frequency (day/year), and *AT* is the time period over which the dose is averaged (day) [15].

For the carcinogenic and non-carcinogenic risks of heavy metals, the carcinogenic risk (*CR*) can be calculated according to Formula (5). In order to assess the carcinogenic risk posed by more than one chemical and/or multiple pathways, the carcinogenic risk index (*CRI*) can be determined according to Formula (6). Similarly, Formula (7) can be used to calculate the non-carcinogenic risk index (*HI*).

(5) CR=ADI×SF

(6) $$CRI=\sum^{\;}ADI_{i}\times SF_{i}$$

(7) $$HI=\sum^{\;}HQ_{i}=\sum^{\;}\frac{ADI_{i}}{RfD_{i}}$$

where *SF* is the slope factor (mg/kg-day), *RfD* is the reference dose (mg/kg-day) [14].

## 3. Results and Discussion

### 3.1. Heavy Metal Contents in Recycled Products

**Figure 1:**
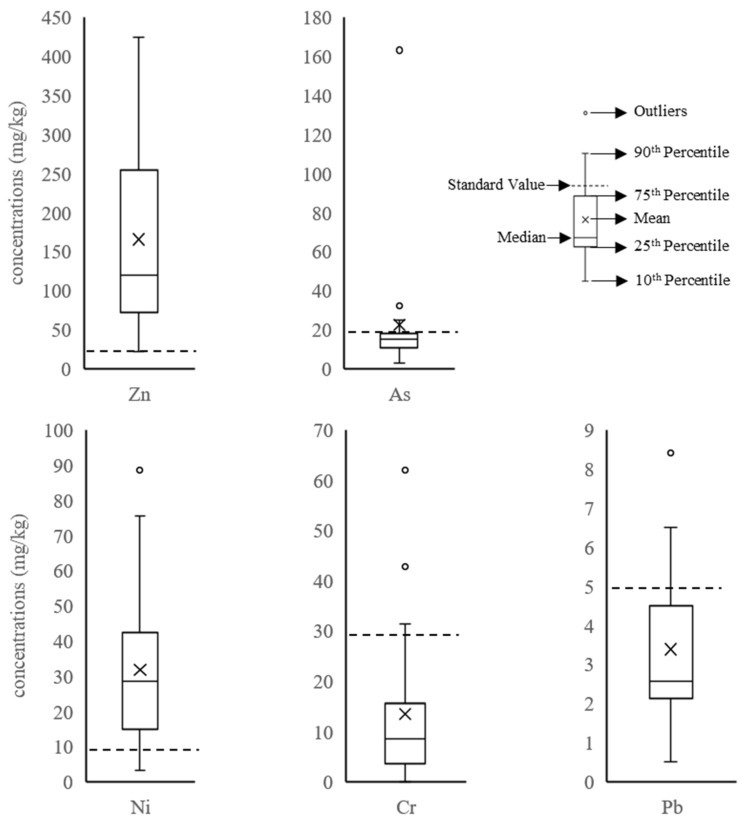
Boxplots of the heavy metal concentrations (mg/kg) for the recycled products.

From Figure 1, the average concentration of each heavy metal in most recycled products exceeds the corresponding product quality standard value. The mean concentrations of Zn, Ni, and As are about 8.3, 3.2, and 1.1 times greater, respectively than the standard values. For Zn, the content of Zn in all samples exceeded the standard value, and the maximum content exceeded the standard value by 21 times. The average contents of Ni and Cr were less than the standard values, while Hg and Cd were not detected. From this analysis, the recycled products were contaminated the most by Zn and the least by Cd and Hg.

**Figure 2:**
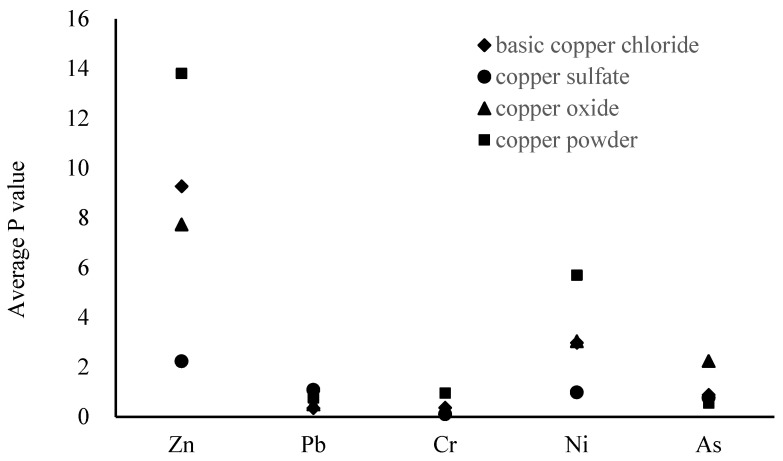
Average *p*-values of heavy metals for different types of recycled products.

As shown in Figure 2, among the recycled products, copper sulfate appears to be the least contaminated, with most of the *p*-values below 0.7. This may be due to the fact that the copper sulfate production process is more conducive to the separation and removal of heavy metals [2]. Meanwhile, basic copper carbonate, copper oxide, and copper powder show the highest *p*-values for heavy metals. The *P*-value of Zn in basic copper carbonate is as high as 9.27, and that of Ni is close to 3. Additionally, the *p*-values of Zn, Ni, and As in copper oxide are all greater than 2, while the *p*-values of Zn and Ni in copper powder are all greater than 5. It should be noted that the *P*-value of Zn in all samples of the four recycled products is greater than 2.

Table 3 reveals the class distribution of *p*-values for heavy metals, which was based on the classification system in Table 2. The results showed that the *p*-values of Pb in all the recycled products were lower than grade 2, and nearly 75% of the products belonged to class 1. More than 85% of the recycled products were lower than class 1 for Cr. Meanwhile, the *p*-values for Zn were above class 4 for nearly 95% of samples. The *p*-values of Ni and As vary the most, ranging from class 1 to class 5; however, the *p*-values of these two elements are mostly lower than class 3.

**Figure 3:**
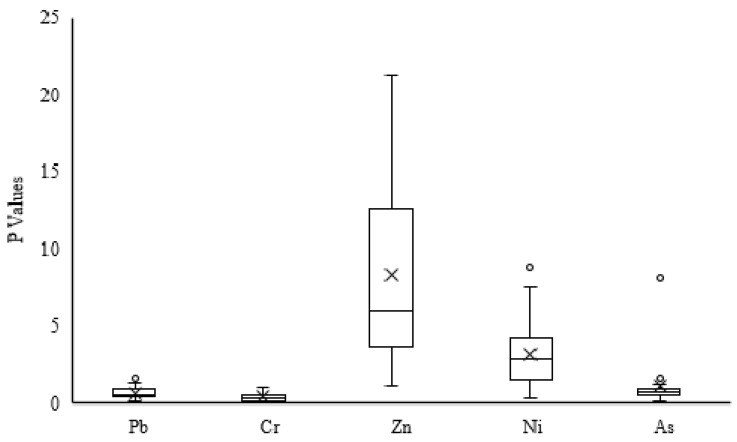
Boxplots of the *p*-values for the heavy metals.

As showed in Figure 3, the average *p*-values for Pb and Cr were 0.68 and 0.45, respectively, classifying these two elements as practically uncontaminated. The average *p*-values of as for all recycled products ranged between 1 and 2, indicating that the samples were marked as moderately contaminated. The average *p*-values for Ni and Zn categorized these metals into class 3. Overall, the contamination levels of these heavy metals are generally in the order of Zn > Ni > As > Pb/Cr > Hg/Cd.

### 3.2. Non-Carcinogenic Risk

Since dermal absorption factors for Cr, Pb, Hg, Ni, and Zn were not available, the non-carcinogenic hazard index of Cr, Pb, Hg, Ni, and Zn were calculated from ingestion and inhalation absorption pathways. For non-carcinogenic risks, ingestion-absorption was the primary exposure pathway for As, Cd Cr, Pb, Hg, Ni, and Zn. For example, the average daily intake of Zn through the ingestion absorption pathway for adults reaches levels of 0.024 mg/kg-day, whereas the average daily intake of Zn through the dermal and inhalation pathway for adults is only 0.0044 mg/kg-day and 0.0002 mg/kg-day respectively.

Of all the heavy metals investigated, people are most exposed to As and Ni due to their high concentrations in the recycled products or low reference doses, while they are least exposed to the remaining heavy metals. For example, the hazard quotients of As and Ni accounted for 87.5% and 9.3% of the entire HI value, respectively. In comparison, the overall proportion of the remaining heavy metals in the overall HI was 3.2%. It is worth mentioning that although the *P*-value of Zn was the highest, the non-carcinogenic environmental risk of Zn only accounted for 0.3% of the total risk.

**Figure 4:**
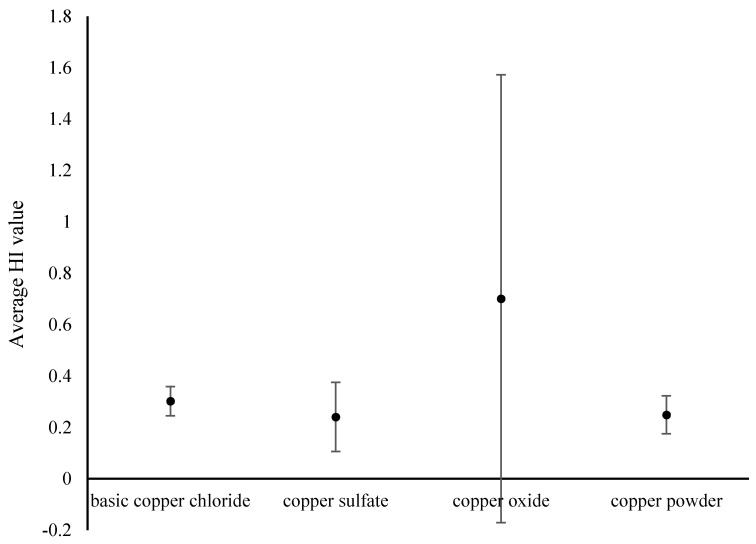
Average HI values (± standard deviation) for different types of the recycled products.

From Wang et al.’s study [16], if the value of HI is less than 1, there are no significant adverse health effects; if the value of HI is greater than 1, non-carcinogenic effects are likely to appear, and the likelihood of such effects increases as the HI value increases. As shown in Figure 4, The HI values of basic copper carbonate, copper sulfate, and copper powder were less than 1, which indicates that the non-carcinogenic risk of these three recycled products was within the acceptable range. However, the HI value of some CuO samples was greater than 1, which indicates that once the recycled product enters the supply chain, it may pose a threat to human health.

### 3.3. Carcinogenic Risk

Owing to the lack of carcinogenic slope factors for other heavy metals, only the carcinogenic risk of As was assessed. In terms of carcinogenic risk, ingestion absorption is the main exposure pathway for As. The average intake of As by the ingestion absorption route in adults reached a level of 0.0037 mg/kg-day, while the average intake of As by the dermal and inhalation routes in adults was only 0.0007 mg/kg-day and 0.00003 mg/kg-day, respectively. This result was analogous to the findings of a study that examined the health risks of heavy metal contamination in the Daxin manganese mining area [17].

In accordance with the work by Sharafi et al. [18], the acceptable carcinogenic risk index ranges between 10^−6^ to 10^−4^. It implies that risks of greater than 10^−4^ are regarded as being unacceptable, while risks of less than 10^-6^ are not perceived as having a substantial undesirable impact. Average As carcinogenic risk values for copper sulfate, copper oxide, basic copper chloride, and copper powder are 1.09 × 10^−5^, 3.19 × 10^−5^, 1.29 × 10^−5^, 7.94 × 10^−6^, respectively. Therefore, the average carcinogenic risk of these four recycled products was within the acceptable range. However, it is worth noting that the maximum carcinogenic risk of copper oxide was 1.16 × 10^−4^, which is unacceptable.

## 4. Recommendation

The existing product quality standards are obviously not suitable for recycled products of spent coppery etchant. For example, the comprehensive index *P*-value of heavy metal Zn is generally high, which indicates that the Zn content in recycled products is significantly higher than the limit value of existing product quality standards. However, the results of the human health risk assessment show that the risk level of Zn in recycled products can be almost negligible. In addition, the As content in some samples is high, which makes the carcinogenic risk and non-carcinogenic risk of these recycled products exceed the acceptable range. However, most product quality standards do not attach importance to As. In addition, Cd and Hg were not detected in these recycled products, indicating that Cd and Hg are not characteristic pollutants. Meanwhile, among the four common recycled products, copper oxide has the highest HI value, while copper sulfate has the lowest environmental risk. Therefore, the management department can strengthen the management of copper oxide, or introduce policies to encourage the production of copper sulfate.

To sum up, it is urgent that a standard is established that comprehensively considers the environmental risk and product quality of recycled products from spent coppery etchant. The formulation of standard limits should consider controlling the environmental risk of recycled products, and should not set the requirements too high to increase unnecessary process costs. According to the carcinogenic and non carcinogenic risk assessment, the recommended limited concentrations for As, Ni, Cr, Pb, and Zn in recycled products are 50–100 mg / kg, 100–300 mg / kg, 50–100 mg / kg, 100–300 mg / kg, 200–500 mg / kg, respectively, and Cd and Hg are not required. If these recycled products can be restricted to industrial use by regulatory means, the corresponding concentration limits can be set higher.

## 5. Conclusions

An investigation of heavy metal pollution in recycled products of spent coppery etchant and a human health risk assessment was conducted for this study. The general situation of heavy metal pollution was firstly described. Zn was the highest content component, however, as pollution was the most momentous in recycled products. Human health risk assessment was calculated based on the risk exposure model recommended by the U.S. EPA, and the results indicated that both non-carcinogenic and carcinogenic risks of heavy metals in recycled products were acceptable. In addition, the leaching rate or stability of heavy metals can be considered in evaluating the risk of heavy metals in recycled products. Despite its limitations, we believe this is the first report about information on recycled products in China. This article may provide some evidence and references for future management decisions.

## Figures and Tables

**Table 1:** The product quality standard value of heavy metals (mg/kg).

Metal	Standard Value ^1^
Ni	10
Cd	6
Cr	30
As	20
Pb	5
Hg	N.A.
Zn	20
Ni	10
Cd	6

^1^ The standard values of heavy metals mainly refer to China’s national or industrial product quality standards and take the minimum value in each standard. N.A. = Not Available.

**Table 2:** The product quality classification.

Class	Value	Product Quality
1	*P* ≤ 0.7	Practically uncontaminated
2	0.7 < *P* ≤ 1	Uncontaminated to moderately contaminated
3	1 < *P* ≤ 2	Moderately contaminated
4	2 < *P* ≤ 3	Moderately to extremely contaminated
5	*P* > 3	Extremely contaminated

**Table 3:** Class distribution of *P*-value for heavy metals.

Class	Zn	Pb	Cr	Ni	As
1	0%	65%	80%	15%	40%
2	0%	15%	5%	0%	40%
3	10%	20%	10%	15%	15%
4	10%	0%	5%	25%	0%
5	80%	0%	0%	45%	5%
